# Predictors and treatment outcome of hyperglycemic emergencies at Jimma University Specialized Hospital, southwest Ethiopia

**DOI:** 10.1186/s13104-015-1495-z

**Published:** 2015-10-11

**Authors:** Tigestu Alemu Desse, Tesfahun Chanie Eshetie, Esayas Kebede Gudina

**Affiliations:** Clinical Pharmacy Department, School of Pharmacy, Jimma University, Jimma, Ethiopia; Departments of Internal Medicine, School of Medicine, Jimma University, Jimma, Ethiopia

**Keywords:** Hyperglycemic emergencies, Predictors, Treatment outcome, Ethiopia

## Abstract

**Background:**

Diabetic ketoacidosis (DKA) and hyperosmolar hyperglycemic state (HHS) commonly known as hyperglycemic emergencies are the two most common life-threatening acute metabolic complications of diabetes. The objective of this study is to assess predictors and treatment outcome of hyperglycemic emergencies (HEs) among diabetic patients admitted to Jimma University Specialized Hospital (JUSH).

**Methods:**

It is a three year retrospective review of medical records of patients admitted with HEs at JUSH. Patient demographics, admission clinical characteristics, precipitants, insulin used and treatment outcomes were extracted. Statistical analysis was done using student’s *t* test, Chi square test, and binary logistic regression with level of α set at 0.05. Statistical significance was considered for variables with p < 0.05.

**Results:**

Complete data was available for 163 out of 421 patients admitted with HEs. The majority (62.6 %) were males. Mean age of patients was 36.6 ± 15.9 years. About 64 % of patients had type 1 diabetes. About 93 % of the participants developed DKA. The most common precipitants of HEs were infections 95 (59 %), non-compliance to medications 52 (32.3 %), and newly diagnosed diabetes 38 (23.6 %). Recurrent hyperglycemia, hypoglycemia and ketonuria occurred in 88 (54 %), 34 (20.9 %) and 31 (20.5 %) patients respectively. Mean amount of insulin used and duration of treatment till resolution of DKA were 136.85 ± 152.41 units and 64.38 ± 76.34 h respectively. The median length of hospital stay was 6 days. Mortality from HEs was 16 (9.8 %). Admission serum creatinine >1.2 mg/dL (P = 0.018), co-morbidity (P < 0.001) and sepsis (P = 0.014) were independent predictors of HEs mortality.

**Conclusions:**

Infections, non-compliance and new onset diabetes were the most common precipitants of HEs. Length of hospital stay and mortality were high. High use of insulin, recurrent hyperglycemia, hypoglycemia, and ketonuria were common during HEs management. Elevated serum creatinine, sepsis and co-morbidity are independent predictors of HEs mortality.

## Background

Diabetes mellitus (DM) is frequently associated with acute non-metabolic and metabolic complications. Diabetic ketoacidosis (DKA) and hyperosmolar hyperglycemic state (HHS), commonly known as hyperglycemic emergencies (HEs), are the two most common life-threatening acute metabolic complications of diabetes [1]. DKA occurs in type 1 and most often HHS occurs in type 2 diabetes; however, each type of diabetes may be associated with DKA or HHS [2]. These complications are usually associated with disability, reduced life expectancy and enormous health costs for virtually every society [3] with mortality rates ranging from 2 to 5 % for DKA and up to 15 % for HHS [4, 5]. In the United States of America, 43 % of the total medical costs for diabetes is spent on hospital inpatient care [6]. The costs of DKA treatment are significant, with estimated mean expenses for a single hospitalization ranging from $7470 to $20,864 [7].

In Ethiopia the prevalence of diabetes is increasing for the last two to three decades becoming a major economic problem in drug use and bed occupancy [8]. The control of diabetes and prevention of ketoacidosis in Ethiopian patients is hampered by socioeconomic factors, particularly the cost and unreliability of insulin supplies [9]. Diabetes is a frequent cause of morbidity and mortality in Ethiopia because of scarce inpatient facilities and limited resources [10].

More than one-third, but less than half, of diabetic patients in Ethiopia receives standard diabetes care [11]. Access for blood glucose monitoring and diabetic education in the country is low resulting in poor control of hyperglycemia often related to poverty and interruption of therapy for many social and economic reasons. For example, 95 % of DM patients don’t perform self-blood glucose monitoring at home, 33 % don’t take their treatments regularly, 75 % require admissions directly or indirectly due to uncontrolled diabetes [12, 13].

The cost of inpatient diabetes management in the country is enormous and significantly higher than the cost of other inpatient managements [8, 10]. DKA is a common cause for diabetic admission in about 71.1 % of diabetic admissions with a mortality of 5.8 % [14]. Among DM patients on follow up at JUSH, only 18.1 % of patients were able to control their fasting blood sugar below 7 mmol/l [15] and DKA was the commonest acute complication and cause of hospital admission [16, 17]. Despite all the challenges and costs incurred by diabetes and its complications, studies are limited on the overall treatment complications, precipitants, length of hospital stay and mortality of acute metabolic diabetes complications (HEs) in the country. Hence forth, this study aimed to assess such problems faced by diabetic patients admitted with HEs and predictors of HEs mortality.

## Research methods and participants

We conducted a retrospective review of the medical records of adolescent and adult diabetic patients with HEs admitted to JUSH from January 01/2011 to December 31/2013. JUSH is a public hospital with 523 beds serving for approximately 20,000 admissions and 140, 000 outpatient visits a year with a catchment population of about 15 million people. Diabetes clinic at the hospital provides twice weekly outpatients services to over 100 diabetic patients a week. Antidiabetic medication(s) dose adjustment and regimen change is made based on patient fasting blood glucose readings on subsequent visits as the hospital does not use glycated hemoglobin (HbA1C) test for regular glucose monitoring of diabetic patients.

Ethical clearance was obtained from Jimma University Collage of Public Health and Medical Sciences Ethical Review Committee to conduct the study. Official letter was obtained from JUSH Clinical Director to access the participants’ medical records for data abstraction. As the study was done on patient medical records retrospectively, no written informed consent and/or ascent for participation in the study were required from participants. However, patient records were handled properly during the data collection process and ethics of research was maintained. Medical record of patients with HEs admitted to the hospital were traced retrospectively in a reverse chronological order from patient logbook and drawn from card room. Selection of medical records for sampling was based on the physician’s confirmed diagnosis on patient logbooks. Participants included in the study were all Diabetic patients with HEs admitted to JUSH with age ≥15 years old whose medical records contained complete pertinent data. Pregnant mothers admitted with HEs were excluded from the study. The data was collected by the Medical Interns of Jimma University students practicing at the hospital.

Data extracted included sociodemographic characteristics, type of diabetes, duration of diabetes and duration of treatment, admission blood pressure, laboratory investigations (admission blood glucose, serum electrolytes, Blood Urea Nitrogen, and Serum Creatinine at presentation), antidiabetic and concomitant medications, precipitants, co-morbidities, amount of insulin used, duration of therapy till HEs resolution and treatment outcomes. Patients with incomplete data such as demographics, admission laboratory tests, treatment interventions and outcomes were excluded. Over a three year period, a total of 421 patients were admitted with HEs of which only 163 patients’ medical records contained complete information and studied. The main outcome of the study was in hospital mortality due to HEs and secondary outcomes were episodes of hypoglycemia during treatment, hyperglycemia and ketonuria after resolution of HEs and Length of hospital stay.

### Statistical analysis

Data was analyzed using SPSS Version 16.0 (Chicago, SPSS Inc.). Student’s *t* test was used for comparison of means of continuous variables. To see the association between categorical variables, Chi square tests or Fisher exact tests were used. To examine independent predictors of mortality, variables with p-values <0.25 on a bivariate binary logistic regression model were entered into a multivariate logistic regression model and variables with p-value <0.05 were considered statistically significant.

### Operational definitions

DKA was defined as admission blood glucose >250 mg/l and urine dipstick ketone level ≥ +2 and HHS was defined as blood glucose >600 mg/dL, alteration in mental status and mild or absent ketonuria. Hyperglycemia was defined as random plasma glucose >200 mg/dL and Hypoglycemia was defined as a blood glucose level <70 mg/dL. Sepsis was defined as the presence of known or suspected infection plus two or more of the following: Temperature >38 or <36 °C, HR >90, RR >24, WBC >12,000/mm^3^ or <4000/mm^3^.

## Results

### Baseline characteristics of patients with hyperglycemic emergencies

A total of 421 diabetic patients were admitted and treated for DKA and HHS at JUSH over a three year period. Of these patients, only 163 of them had complete data and were included in the study. Hundred-two (62.60 %) of them were males. The mean age of the participants was 36.57 ± 15.91 (range 15–84 years). About 48 % of the participants were below 35 years old. More than half of the patients (56.4 %) were from urban and the remaining 74 (43.6 %) were rural residents. Family history of diabetes was reported in only 2 of the patients. The majority, 104 (63.8 %), of the participants had type 1 diabetes. All type 1 and 47 (79.7 %) of type 2 diabetic patients developed DKA while HHS was noted in type 2 diabetics (P < 0.001). Overall, there were 151 (92.6 %) cases of DKA and 12 (7.4 %) cases of HHS. All HHS cases were seen in patients older than 34 years.

Seventy four (45.4 %) of the patients were newly diagnosed with diabetes at admission and the remaining 89 (54.6 %) were known diabetic patients on follow up at JUSH diabetic clinic. Eighty five (95.5 %) of the known diabetic patients developed DKA. Eight (66.7 %) out of 12 who developed HHS were new DM patients. The majority, 94 (57.7 %), of the participants had diabetes for less than a year while only 6 (3.7 %) patients had diabetes for more than 10 years. Ninety five (58.3 %) of the study subjects were on antidiabetic medications for <1 year. No HHS patient had either diabetes or was on antidiabetic medications for more than 5 years.

A total of 87 (53.4 %) patients were on antidiabetic medications prior to admission out of which 58 (66.7 %) were on insulin and 26 (29.9 %) on oral glucose lowering agents (OGLAs). The remaining 3 patients were on insulin + OGLAs. Out of the 29 patients who were on OGLAs, 8 (27.6 %) were on Glibenclamide, 9 (31 %) were on Metformin and 12 (41.4 %) were on Glibenclamide + Metformin. All patients who were on insulin developed DKA (P = 0.005). Eighty three (95.4 %) of the patients who were on antidiabetic medications developed DKA. Thirteen (8.0 %) out of 163 patients had been taking concomitant medications prior to admission. There were 36 (22.1 %) patients with co-morbidities of which 15 (41.7 %) had hypertension, 7 (19.5) had cardiovascular disease, 6 (16.7 %) had stroke and the remaining had viral hepatitis, chronic kidney disease and malignancies. Eleven patients (6.7) had history of hospital admission due to HEs within the past 12 months.

Hypotension (BP <90/60 mmHg) on admission was noted in 8 (4.9 %) of the patients. Hypokalemia (serum K^+^ <3.5 mEq/L) and hyponatremia (Serum Na^+^ <135 mEq/L) at admission were observed in 37 (22.7 %) and 48 (29.4 %) of the patients respectively. There were 34 (20.9 %) patients with elevated admission serum creatinine (>1.2 mg/dL). The Mean admission blood glucose was 464.93 ± 99.81 mg/dL. Mean durations of diabetes and antidiabetic treatment were 2.42 ± 4.02 and 2.36 ± 3.96 years respectively. Most of the patients 131 (80.4 %) had admission GCS = 15 and the remaining 32 (19.6 %) had admission GCS <15. Admission urine ketone for DKA patients ranged from +2 to +4 with preponderance of +2 level in 81 (49.7 %) of the patients.

Admission characteristics of DKA and HHS patients are compared in Table 1. Patients with HHS were significantly older than those with DKA (56.25 ± 8.68 vs. 35.00 ± 15.31 years, P < 0.001).

**Table 1:** Admission characteristics of diabetic patients by type of HE at JUSH

Parameter	DKA (Mean ± SD)	HHS (Mean ± SD)	P-value
Age (years)	35.00 ± 15.31	56.25 ± 8.68	<0.001*
Blood glucose (mg/dL)	454.20 ± 95.83	>600.00	<0.001*
SBP (mmHg)	113.61 ± 23.64	95.83 ± 33.97	0.017*
DBP (mmHg)	74.44 ± 13.68	62.50 ± 22.21	0.006*
Serum potassium (mEq/L)	3.78 ± 0.47	3.70 ± 0.57	0.591
Serum sodium (mEq/L)	137.71 ± 4.52	135.46 ± 7.17	0.116
Serum chloride (mEq/L)	109.67 ± 7.42	107.79 ± 6.49	0.395
Serum creatinine (mg/dL)	1.18 ± 0.99	1.63 ± 0.97	0.135
GCS	14.52 ± 1.61	14.00 ± 1.21	0.273

*SBP* systolic blood pressure, *DBP* diastolic blood pressure, *BUN* blood urea nitrogen, *GCS* Glasgow Coma Scale

* Statistically significant

### Precipitants of hyperglycemic emergencies

Precipitating factors of HEs were determined for 161 (98.8 %) of the patients. The most common precipitants of HEs were infections 95 (59 %), non-compliance to antidiabetic medications 52 (32.3 %) and newly diagnosed diabetes 38 (23.6 %). The major cause of non-compliance was medication discontinuation which was reported in 45 (86.5 %) of the patients. Urinary tract infection 61 (64.2 %) was the most common infection that precipitated HEs (Table 2).

**Table 2:** Precipitants of HEs of diabetic patients admitted to JUSH

Precipitating factor	Frequency (%) n = 161
Infection	95 (59.0)
Non-compliance	52 (32.3)
Newly diagnosed DM	38 (23.6)
Trauma (injury)	1 (0.6)

Precipitants by type of non-compliance and infection	Frequency (%) n = 161
**Non-compliance to medication(s)**	
Medication(s) discontinuation	45 (86.5)
Missed dose of medication	7 (13.5)

Type of infection	Frequency (%) n = 161
UTI	61 (64.2)
Pneumonia	13 (13.7)
Sepsis	10 (10.5)
Pulmonary TB	4 (4.2)
Oral infections	3 (3.2)
Insulin injection site infection	3 (3.2)
DFU	3 (3.2)
Osteomyelitis	2 (2.1)
Gastrointestinal infection	2 (2.1)
Bacterial meningitis	2 (2.1)
Others	13 (13.7)

*UTI* urinary tract infection, *TB* tuberculosis, *DFU* diabetic foot ulcer

*NB* total percentage is >100 % as there were multiple response questions

### Treatment interventions and outcomes of patients with HEs

Mean amount of insulin used (units) till resolution of DKA was 136.85 ± 152.41 whereas 71.83 ± 33.29 units of insulin were used till HHS resolution. The durations of treatment till resolution of DKA and HHS were 64.38 ± 76.34 and 29.00 ± 20.58 h respectively. The majority, 88 (54 %), of patients experienced one or more episodes of hyperglycemia after resolution of HEs. Episodes of hypoglycemia were noted in 34 (20.9 %) of the patients (Table 3). The overall in hospital mortality due to HEs among the study subjects was 16 (9.8 %).

**Table 3:** Frequency of metabolic complications and prognosis of diabetic patients with HEs admitted to JUSH

Parameter	DKA	HHS	Total	P-value
	Frequency (%)	Frequency (%)	Frequency (%)	
Episodes of hyperglycemia	82 (93.2)	6 (6.8)	88 (54.0)	1.00
Episodes of hypoglycemia	33 (97.1)	1 (2.9)	34 (20.9)	0.463
Episodes of ketonuria (DKA)	31 (100.0)	–	31 (20.5)	0.125
Died	15 (9.9)	1 (8.3)	16 (9.8)	1.00
Improved and discharged	135 (92.5)	11 (7.5)	146 (89.6)	1.00
Left against medical advice	1 (100.0)	0	1 (0.6)	0.074

The median length of hospital stay was 6 days; 7 days for DKA patients and 5 days for HHS ones. The large majority, 87 (53.4 %), of patients had length of hospital stay 1–7 days. Ten (6.1 %) patients stayed at the hospital for more than 28 days. Most of the deaths, 14 (87 %), occurred within the first seven days of admission (p = 0.011).

Mortality rate in males was higher than in females (11.8 vs. 6.6 %), however, it was not statistically significant (P = 0.286,). The highest mortality rate was observed in age groups ≥65 years, 3 (37.5 %) whereas the lowest mortality rate occurred in 15–24 age groups, 1 (2.5 %). The number of deaths in type 1 DM and type 2 DM patients was equal (8 in each). Mortality among known DM patients, 13 (14.6 %), was higher than among new ones 3 (4.1 %); P = 0.035. The highest mortality, 8 (30.8 %), was recorded in patients with 6–10 years duration of diabetes and antidiabetic treatment (P < 0.001). No death was recorded in patients with durations of diabetes and antidiabetic treatment above 10 years. Six (46.2 %) out of 13 patients taking concomitant medications were died (P < 0.001) and 12 (33.3 %) patients died out of 36 who had co-morbidity (P < 0.001).

On a multivariate model (Table 4), independent predictors of mortality were elevated admission serum creatinine >1.2 mg/dL (AOR = 5.86, 95 % CI: 1.36–25.28, P = 0.018), sepsis (AOR = 9.83, 95 % CI: 1.59–60.79, P = 0.014) and co-morbidity (AOR = 15.26, 95 % CI = 3.67–63.41, P < 0.001).

**Table 4:** Independent predictors of HEs related mortality of diabetic patients admitted to JUSH

Variable category	Died		P-value	AOR^a^ (95 % CI)
	Yes (n)	No (n)		
History of DM				
New DM	3	71		1
Known DM	13	76	0.057	4.88 (0.95–24.97)
Serum creatinine (mg/dL)				
<0.5	0	2	0.999	0 (0)
0.5–1.2	8	119		1
>1.2	8	26	0.018	5.86 (1.36–25.28)
Co-morbidity				
No	4	123		1
Yes	12	24	<0.001	15.26 (3.67–63.41)
Sepsis				
No	10	143		1
Yes	6	4	0.014	9.83 (1.59–60.79)

^a^ *AOR* adjusted odds ratio

## Discussion

Our study showed that DKA was the most common diagnosis among diabetic patients with HEs and infection was the leading precipitating factor of HEs admission. Metabolic complications during treatment were common. Lengths of hospital stay and in hospital mortality of HEs were high. Elevated admission serum creatinine, sepsis and co-morbidity were found to be independent predictors of HEs mortality.

We found that DKA and HHS occurred in 92.6 and 7.36 % diabetic patients with HEs respectively. In this study the proportion of DKA patients was higher than the findings from other studies, while that of HHS was lower than the same findings [18–20].

The reasons for the higher proportion of DKA in our finding may be due to the combined effect of: (1) DKA was common in type 1 diabetes where the majority of patients in our study are type 1 diabetics, (2) there are reports [21] that DKA is common in known diabetics and the majority of our patients were known diabetics and on antidiabetic treatment who were non-compliant to their medications (52 out of 87 patients that were on treatment were non-compliant), (3) most of the patients were in an actively growing and working age group (15–44 years) whose insulin demand may fluctuate based on their daily activities that need dose adjustment based on demand and (4) high infection rate recorded as the most common precipitant which may increase insulin demand leading to ketoacidosis.

Admissions for HHS, in contrast to DKA, were more common in patients with a new diagnosis of diabetes (66.7 %). This is in line with the finding from South Africa [22], but against the study from Taiwan [23] where HHS was more common in known DM patients than newly diagnosed ones (72.3 vs. 27.7 %).

The higher incidence of HHS in new DM patients in our finding and the similarity to the finding from South Africa may be related to relatively older age of patients with HHS (91.67 % of HHS patients were ≥45 years in our study whereas all patients were >40 years old in the South African study) where hyperosmolarity is usually related to an age-related increase in the renal threshold for glucose and reduced sensitivity of the thirst centre. It is also possible that as HHS admissions were mainly of newly diagnosed diabetic subjects who were not aware of their diabetes status, they may have presented relatively late due to delayed recognition of hyperglycemic symptoms or not taken adequate fluids to replenish ongoing fluid losses (which an informed known diabetic patient could do so). It also suggests that many adult patients remain undiagnosed of diabetes and present for medical attention after developing diabetic complications and worsening of the conditions.

The most common precipitants of HEs in our study were infections 59 %, non-compliance to antidiabetic medications 32.3 % and newly diagnosed DM 23.6 %. These values are higher than the findings from Pakistan and South Africa [24, 25]. However, a higher rate of infection than our finding was reported from Thailand [26]; 73.5 vs. 59 %.

Poor infection prevention strategies by the patients either due to poor patient awareness or the underlying poverty might have contributed to the higher rate of infection in our study. It is also possible that about 45 % of the patients is newly diagnosed at admission whose awareness of self care and infection prevention may be low. There is a report [27] that, in Sub Saharan Africa, healthcare systems are scarce due to widespread poverty of individuals and nations alike calling for the need for empowerment of individuals and populations through education, nutrition and poverty eradication to improve self-care in health and living with diabetes.

The rate of non-compliance in our finding was lower than the findings from America, Thailand, Nigeria and Kenya that range from 34 to 69 % [18, 26, 28–30]. This may be attributed to the high number of patients with no prior treatment (46.6 %) which might have contributed to the decrease in total percentage of non-compliance rate. Otherwise, this non-compliance rate is not acceptable and hence patient education, improvement in accessibility of nearby health facilities that provide medical care for diabetics and economic empowerment of DM patients may be warranted. It has been reported [27, 31] that stopping insulin for economic reasons, unavailability and unaffordability of insulin, missed clinics, perceived ill-health and alternative therapies like herbs, prayers and rituals are common problems that contributed to non-compliance to antidiabetic medications in the Tropical and Sub-Saharan African diabetic patients.

We found that the mean amount of insulin used till resolution of DKA and HHS was 136.85 ± 152.41 and 71.83 ± 33.29 units respectively. The mean amount of insulin used till resolution of DKA in our study was far higher than the mean amounts of insulin used in the studies from America [32–34]. The mean duration of treatment till resolution of DKA in our study was more than five times (64.38 ± 76.34 h) the time needed in other studies [32–34]. The higher amount of insulin use and longer duration of treatment until DKA resolution as compared to other studies can be explained by; firstly, the difference in the management protocol of DKA between ours and other studies. Other studies used continuous intravenous infusion of regular insulin at high care centers (intensive care units) to manage DKA while in our study they were treated at general medical wards with sliding scale insulin. Secondly, the pharmacokinetic difference between intramuscular (IM) and/or subcutaneous (SC) and continues intravenous infusion of regular insulin. Thirdly, in our study DKA is said to be resolved based on blood glucose, urine dipstick ketone clearance and clinical parameters while in other studies it is based on blood glucose, PH and serum bicarbonate levels which are more objective than ours. In addition, the commitment of multidisciplinary team to monitor patients closely and administer hourly IM (SC) insulin injection, severity of DKA at presentation, and the presence of co-morbidities might have contributed for this difference. The use of SC insulin injections on an hourly schedule is reported to be difficult to follow in many medical centers because of the intensity of treatment and shortage of nursing staff in general wards [33, 34].

Fisher et al. [35] reported that, in DKA patients treated either with IM or SC injections or with continuous IV infusion of regular insulin, 30–40 % of patients in the IM and SC groups did not lower their plasma glucose by 10 % in the first hour after insulin injection and that the concentration of ketone bodies was lowered at a significantly faster rate in the IV group than with IM or SC insulin. The delay in onset of action of regular insulin was supported by the report of Menzel and Jutzi [36], who treated patients with frequent small SC injections, but only 4 of 24 patients showed a fall in blood glucose concentration in the first 3 h of therapy. It was also reported that the mean hourly rate of fall of plasma glucose level was significantly higher in patients treated with intravenous insulin therapy group than patients treated with intramuscular insulin therapy [37]. These differences in response can be explained by delays in reaching a therapeutic plasma insulin concentration to produce the desired response.

The median length of hospital stay was 6 days range from 1 to 59 days. This finding was far lower than the finding from the study by Ezeani et al. [20] and higher than other studies [3, 38]. The reason for the longer duration of hospital stay in our finding is unclear and probably further research may be needed. The reason for a shorter duration of hospital stay in our finding may be related to initial patient presentation. For example, DFU was common in the study we compared whereas only 3 patients had DFU in our study. It was reported that [20, 38] DFU is usually associated with a much longer duration of hospital stay.

In our finding episodes of hyperglycemia (54.0 %) was lower than the report from Thailand [26] while hypoglycemic episodes (20.9 %) in our study were higher than the same study in Thailand (15.7 %). Though we are uncertain to explain the noted difference in hyperglycemic episodes between our patients and the Thailand’s, it might possibly be related to the difference in patient characteristics at presentation as infection and admission blood glucose were higher than ours. This may be substantiated by the reports from Queale et al. [39] where severity of illness, severe diabetic complications, high admission glucose level and admission for infectious disease were found to be independent predictors of hyperglycemic episodes. The higher hypoglycemic episodes documented in our finding may be related to infrequent patient blood glucose and urine dipstick ketone monitoring to adjust insulin doses based on patient need that may lead to inadvertent use of insulin resulting in hypoglycemia.

We noted that episodes of hypoglycemia among DKA patients were 21.9 %. This figure is higher than other studies that reported 5 and 6.7 % [33, 34] respectively and lower than the reports from Guillermo et al. [32]. While the higher hypoglycemic events in our study might be due to the differences in DKA management protocol and set up and infrequent laboratory monitoring in our set up, the reasons for lower hypoglycemic events may need further investigation.

The overall mortality of HEs in this study was 9.8 %. This is higher than other studies [19, 20, 26, 38, 40] that reported mortality rates ranging from 3.57 to 8.4 % and lower than the findings from Nigeria [18] and Kenya [30]. The higher mortality rate in our finding may be explained by the difference in inpatient management protocol of HEs, presenting precipitants, age, co-morbidities, and absence of adequate laboratory investigations at presentation, inadequate laboratory monitoring during treatment to monitor patient response. The lower mortality rate in our finding as compared to the Nigeria and Kenya studies possibly lays on the difference in patient characteristics.

The majority of deaths (81.2 %) occurred in known DM patients (P = 0.047) and all deaths were documented in patients with <11 years of diabetes duration prior to presentation. This is comparable with the findings by Ogbera et al. [18] and Ezeani et al. [20] who reported that (78.2 %) and all the mortalities respectively were in subjects who had diabetes duration of less than 10 years prior to presentation. This may be due to ignorance of diabetes complications and the need for early presentation to the hospital when complications arise.

Age above 65 years old, previously known DM, DM duration 6–10 years, antidiabetic treatment for 6–10 years, SBP >159 mmHg, DBP >99 mmHg, SeCr >1.2 mg/dL, concomitant medications, co-morbidity, and sepsis are found to have a significant association with HEs related mortality on a bivariate logistic regression model; however, only elevated admission serum creatinine (>1.2 mg/dL), co-morbidity, and sepsis are independent predictors of HEs related mortality on a multivariate logistic regression model.

## Conclusions and recommendations

In conclusion, our study revealed that DKA is the index presentation of HEs. Infections, non-compliance to antidiabetic medications and new onset diabetes were the most common precipitants of HEs. The amount of insulin required and the time needed till resolutions of DKA were found to be high. Recurrent hyperglycemia, hypoglycemia and ketonuria were common problems seen during the management of HEs. Length of hospital stays and mortality rate from HEs were abnormally high. Elevated admission serum creatinine, co-morbidity and sepsis are independent predictors of HEs mortality. Therefore, promoting self-monitoring of blood glucose, patient education on diabetes self-care practices and infection prevention strategies, developing evidence based HEs treatment protocol, organizing HEs managing multidisciplinary team and availing biochemical tests at affordable costs may help prevent hyperglycemic related admissions and unwanted treatment outcomes. Treating physicians should also detect and manage precipitants of HEs and co-morbidities early at initial patient presentation. At last, we recommend large scale multicenter prospective studies to assess the limitations in HEs management in Ethiopians and to devise strategies for cost-effective and evidence-based treatment.

## Abbreviations

BUN: blood urea nitrogen
DBP: diastolic blood pressure
DFU: diabetic foot ulcer
DKA: diabetic ketoacidosis
DM: diabetes mellitus
GCS: glasgow coma scale
HEs: hyperglycemic emergencies
HHS: hyperosmolar hyperglycemic state
JUSH: Jimma University Specialized Hospital
OGLAs: oral glucose lowering agents
SD: standard deviation
SBP: systolic blood pressure
SeCr: serum creatinine
